# Enhancing the quality and transparency of systematic reviews

**DOI:** 10.25100/cm.v49i4.4248

**Published:** 2018-12-30

**Authors:** Herney Andrés García-Perdomo

**Affiliations:** 1 Associate Professor. Universidad del Valle Escuela de Medicina . Grupo de Investigación UROGIV . Cali, Colombia

Systematic reviews (SR) have been important tools for determining the magnitude of an effect, with appropriate methodology, rigor and scientific quality ^1^ ^-^ ^3^. This epidemiologic design was developed to conduct an exhaustive, systematic and explicit assessment of the literature, based on a clearly research question, an explicit methodology, a critical appraisal using a variety of tools and a qualitative summary of the evidence ^3^. On the other hand, the meta-analysis (MA), is the statistical analysis used in the synthesis of the evidence at the end of a very well performed systematic review ^3^. It compares head to head interventions, however nowadays, we have another tool to perform indirect or mixed comparisons (Network meta-analysis) ^4^ ^,^ ^5^. This new statistical tool evaluates the effectiveness when comparing different treatments with similar characteristics, which have not been directly compared in a study. Unlike the traditional meta-analysis, this new tool compares the results of different studies that have a point or a common intervention without a direct comparison ^5^ ^-^ ^7^.

Nowadays, when attending patients, professionals need high-quality evidence to base their practice, however, the number of studies is increasing exponentially and It might be very difficult keeping pace with and assessing the evidence presented. At the same time, SRs / MA are growing in number but not always in quality, comprising a number of ways in which bias can be introduced in SRs such as: 1) Inappropriate methodological quality of primary studies; 2)Publication bias (Statistically significant results and those in English language are more likely to be published); 3) Inclusion criteria influenced by the most favorable outcome and the results of the primary studies; 4) inappropriate statistical approach; among others bias ^8^ ^,^ ^9^ that need to carefully assess in order to make good decisions in clinical and population settings.

The intention of this editorial is to show some key notes, relevant to evidence synthesis that can be applied by any health-related professionals and researchers. 

## Steps to follow in Systematic reviews

The synthesis of the evidence gathers a methodologically correct design that requires a working group and a protocol to be developed.

All protocols for systematic reviews must be written according to PRISMA-P ^10^ and registered in recognized database, for example in the international prospective register of systematic reviews (PROSPERO) (https://www.crd.york.ac.uk/prospero/) from the University of York and the National Institute for Health Research. This allows to be transparent regarding the methods and purpose of this important kind of research.

People who want to perform systematic reviews or to use information in clinical or field settings must adhere to the following requirements outlined below, in order to achieve consistency and comparability ^3^ ^,^ ^11^:

- Establish a clear and concise research question.
- Configure a reproducible search strategy (no limited neither to one language nor to one database).
- Locate and select studies (Published and unpublished).
- Extract the data.
- Assess the quality of the evidence according to the kind of study (Cochrane risk of bias tool, Newcastle - Ottawa Scale (NOS), MINORS, ROBINS-I, QUADAS2 and Grading of Recommendations Assessment, Development and Evaluation (GRADE), among others).
- Analyze and describe the results.
- Perform MA if it is appropriate (fixed or random effects, meta-regression, network meta-analysis, heterogeneity assessment and sensibility).
- Write the manuscript according to PRISMA ^12^.

## Assessing the quality of systematic reviews

Additional to these important issues, health professionals must assess the quality of these manuscripts in order to apply to their patients or analyze in a journal club. This is a fundamental issue to deal with when interpreting the evidence, since it is needed that readers balance the numerical results against the quality of the study to accept the recommendations stated.

Important and standardized tools have been developed to both critically appraise and adequately reporting systematic reviews. These approaches lead to be transparent in science, nonetheless, good reporting (PRISMA statement) is not synonymous to high methodological quality. Two of the most worldwide used tools are: 1)Assessing the methodological quality of systematic reviews (AMSTAR2) ^13^ and 2)Critically Appraisal Skills Programme (CASP) (https://casp-uk.net/). Either one must be used in order to critically appraise systematic reviews in clinical or academic settings as previously stated. 

Furthermore, according to Taylor *et al.* ^14^, ten questions described in Table 1, are one easy way to critically appraise this kind of study, which contains information regarding: The question, the appropriateness of the design, the methods, the statistical analysis and the conflicts of interests**.**

**Table 1:** Ten questions to easily assess systematic reviews.

1. Is the study question relevant?
2. Does the study add anything new?
3. What type of research question is being asked?
4. Was the study design appropriate for the research question?
5. Did the study methods address the most important potential sources of bias?
6. Was the study performed according to the original protocol?
7. Does the study test a stated hypothesis?
8. Were the statistical analyses performed correctly?
9. Do the data justify the conclusions?
10. Are there any conflicts of interest?

Lastly but not the less important, readers need to consider the effect that the author´s conflict of interest might add to the effect size. It is important to elucidate whether the research has industry influence regarding the arguments assessed for policy decision-making, since conflict of interest are frequently inappropriately described in editorials, comments, letters, perspectives and obviously also in systematic reviews. 
