# Supplementary material for: Enhancing the quality and transparency of systematic reviews
Source: Colomb Med (Cali). 2018 Dec 30;49(4):251–3. doi: 10.25100/cm.v49i4.4248 (PMC6342088; doi:10.25100/cm.v49i4.4248)
Supplement: Related texts

# Editorial: Mejorando la calidad y transparencia de las revisiones sistemáticas

Las revisiones sistemáticas (RS) han sido herramienta importante para determinar la magnitud de un efecto, con una apropiada metodología, rigor y calidad científica ^1^^-^^3^. Este diseño epidemiológico fue desarrollado para llevar a cabo una evaluación exhaustiva, sistemática y explícita de la literatura, sobre la base de una pregunta de investigación clara, una metodología explícita, una valoración crítica usando una variedad de herramientas y un resumen de la evidencia cualitativa ^3^ . Por otro lado, el meta-análisis (MA), es el análisis estadístico utilizado en la síntesis de la evidencia, al final de una revisión sistemática muy bien realizada ^3^. Compara intervenciones directas, sin embargo, en la actualidad, tenemos otra herramienta para realizar comparaciones indirectas o mixtas (meta-análisis en red) ^4^^,^^5^. Esta nueva herramienta estadística evalúa la efectividad cuando se comparan diferentes tratamientos con características similares, que no se han comparado directamente en un estudio. A diferencia del meta-análisis tradicional, esta nueva herramienta compara los resultados de diferentes estudios que tienen un punto o una intervención común sin una comparación directa ^5^^-^^7^. 

Hoy en día, al atender pacientes, los profesionales necesitan evidencia de alta calidad para basar su práctica, sin embargo, el número de estudios está aumentando exponencialmente y puede ser muy difícil mantener el mismo ritmo evaluando la evidencia presentada. Al mismo tiempo, las RS/MA están creciendo en número pero no siempre en calidad, comprendiendo varias formas en las que se puede introducir un sesgo en las RS, tales como: 1) Calidad metodológica inadecuada de los estudios primarios; 2) Sesgo de publicación (es más probable que se publiquen resultados estadísticamente significativos y aquellos en idioma inglés); 3) Criterios de inclusión influenciados por el desenlace más favorable y los resultados de los estudios primarios; 4) Enfoque estadístico inapropiado; entre otros sesgos ^8^^,^^9^ que se deben evaluar cuidadosamente para tomar buenas decisiones en entornos clínicos y poblacionales. 

La intención de este editorial es mostrar algunas notas clave, relevantes para la síntesis de evidencia que pueda ser aplicado por cualquier profesional e investigador relacionado con la salud. 

## Pasos a seguir en las revisiones sistemáticas. 

La síntesis de la evidencia recoge un diseño metodológicamente correcto que requiere un grupo de trabajo y un protocolo a desarrollar. 

Todos los protocolos para revisiones sistemáticas deben estar escritos de acuerdo con PRISMA-P ^10^ y registrados en una base de datos reconocida, por ejemplo en el registro prospectivo internacional de revisiones sistemáticas (PROSPERO) (https://www.crd.york.ac.uk/prospero/) de la Universidad de York y el Instituto Nacional de Investigación en Salud. Esto permite ser transparente con respecto a los métodos y el propósito de este importante tipo de investigación. 

Las personas que desean realizar revisiones sistemáticas o utilizar información en entornos clínicos o de campo deben cumplir con los siguientes requisitos que se detallan a continuación para lograr coherencia y comparabilidad ^3^^,^^11^:

- Establecer una pregunta de investigación clara y concisa.
- Configurar una estrategia de búsqueda reproducible (no limitado a un idioma ni a una base de datos).
- Localizar y seleccionar estudios (publicados y no publicados) .
- Extraer los datos.
- Evaluar la calidad de la evidencia según el tipo de estudio (Herramienta de riesgo de sesgo de Cochrane, Escala de Newcastle - Ottawa (NOS), MINORS, ROBINS-I, QUADAS2 y GRADE, entre otros).
- Analizar y describir los resultados.
- Realizar MA si es apropiado (efectos fijos o aleatorios, metarregresión, meta-análisis en red, evaluación de heterogeneidad y sensibilidad).
- Escribir el manuscrito según PRISMA ^12^.

## Evaluación de la calidad de las revisiones sistemáticas. 

Además de estos temas importantes, los profesionales de la salud deben evaluar la calidad de estos manuscritos para aplicarlos a sus pacientes o analizarlos en un club de revistas. Este es un tema fundamental al tratar de interpretar la evidencia, ya que es necesario que los lectores equilibren los resultados numéricos con la calidad del estudio para aceptar las recomendaciones formuladas. 

Importantes herramientas estandarizadas se han desarrollado, tanto para evaluar críticamente como para informar adecuadamente las revisiones sistemáticas. Estos enfoques llevan a ser transparentes en la ciencia, sin embargo, una buena información (Declaración PRISMA) no es sinónimo de alta calidad metodológica. Dos de las herramientas más utilizadas en todo el mundo son : 1) Evaluación de la calidad metodológica de las revisiones sistemáticas (AMSTAR2) ^13^ y 2) Programa de habilidades de evaluación crítica (CASP) (https://casp-uk.net/). Cualquiera de los dos debe usarse para evaluar críticamente las revisiones sistemáticas en entornos clínicos o académicos como se indicó anteriormente. 

Además, de acuerdo con Taylor *et al*. ^14^, 10 preguntas descritas en la Tabla 1, son una manera fácil de evaluar críticamente este tipo de estudio, dado que contiene información sobre: La pregunta, la adecuación del diseño, los métodos, el análisis estadístico y los conflictos de interés**.**

**Tabla 1:** Diez preguntas para evaluar fácilmente revisiones sistemáticas.

1. ¿Es relevante la pregunta de estudio?
2. ¿Agrega el estudio algo nuevo?
3. ¿Qué tipo de pregunta de investigación se está haciendo?
4. ¿Fue el diseño del estudio apropiado para la pregunta de investigación?
5. ¿Los métodos de estudio abordaron las fuentes potenciales de sesgo más importantes?
6. ¿Se realizó el estudio según el protocolo original?
7. ¿El estudio prueba una hipótesis declarada?
8. ¿Los análisis estadísticos se realizaron correctamente?
9. ¿Los datos justifican las conclusiones?
10. ¿Hay algún conflictos de intereses?

Por último, pero no menos importante, los lectores deben considerar el efecto que los conflictos de intereses del autor pueden agregar al tamaño del efecto. Es importante aclarar si la investigación tiene influencia en la industria con respecto a los argumentos evaluados para la toma de decisiones, ya que los conflictos de intereses con frecuencia se describen de manera inadecuada en editoriales, comentarios, cartas, perspectivas y, obviamente, también en revisiones sistemáticas.
